# A Robust Co-Localisation Measurement Utilising Z-Stack Image Intensity Similarities for Biological Studies

**DOI:** 10.1371/journal.pone.0030632

**Published:** 2012-02-17

**Authors:** Yinhai Wang, Craig Ledgerwood, Claire Grills, Denise C. Fitzgerald, Peter W. Hamilton

**Affiliations:** 1 Centre for Cancer Research and Cell Biology, Queen's University Belfast, Belfast, United Kingdom; 2 Centre for Infection and Immunity, Queen's University Belfast, Belfast, United Kingdom; University of California, Berkeley, United States of America

## Abstract

**Background:**

Co-localisation is a widely used measurement in immunohistochemical analysis to determine if fluorescently labelled biological entities, such as cells, proteins or molecules share a same location. However the measurement of co-localisation is challenging due to the complex nature of such fluorescent images, especially when multiple focal planes are captured. The current state-of-art co-localisation measurements of 3-dimensional (3D) image stacks are biased by noise and cross-overs from non-consecutive planes.

**Method:**

In this study, we have developed Co-localisation Intensity Coefficients (CICs) and Co-localisation Binary Coefficients (CBCs), which uses rich z-stack data from neighbouring focal planes to identify similarities between image intensities of two and potentially more fluorescently-labelled biological entities. This was developed using z-stack images from murine organotypic slice cultures from central nervous system tissue, and two sets of pseudo-data. A large amount of non-specific cross-over situations are excluded using this method. This proposed method is also proven to be robust in recognising co-localisations even when images are polluted with a range of noises.

**Results:**

The proposed CBCs and CICs produce robust co-localisation measurements which are easy to interpret, resilient to noise and capable of removing a large amount of false positivity, such as non-specific cross-overs. Performance of this method of measurement is significantly more accurate than existing measurements, as determined statistically using pseudo datasets of known values. This method provides an important and reliable tool for fluorescent 3D neurobiological studies, and will benefit other biological studies which measure fluorescence co-localisation in 3D.

## Introduction

In immunohistochemical (IHC) analysis of biological tissue/cells, co-localisation measurements are widely used to determine whether fluorescently labelled cells, proteins or molecular conformations share an identical or proximal location [1], [2]. This allows determination of whether molecules are expressed in the same cells, are expressed in the same location of a cell or even if two cells are very closely interacting.

A number of quantitative co-localisation measurements are generally used. One such measurement is the Pearson's correlation coefficient [3], however, it is prone to error particularly for partial co-localisation and exclusion [4] resulting in difficulty with interpretation. The overlap coefficient [5] can be strongly influenced by the ratio of the number of foreground objects from each of the two colour channels, whilst also being sensitive to the absolute image intensity values. The most popular coefficient for co-localisation measurements is Manders' co-localisation coefficient [5]. If we define the term “background” to be the captured image regions which appear to be black coloured throughout this paper, the Manders' co-localisation coefficient is, however, very sensitive to how background is removed and is unable to differentiate cross-over situations (random overlapping) from co-localisation, which in turn leads to high incidences of false positives. Many modifications and improvements were proposed over the last two decades, such as the use of image restoration to improve co-localisation analysis [6], [7], and the use of the improved spatial image cross-correlation spectroscopy (ICCS) [8], [9], [10]. Recently described co-localisation measurements are also used by some researchers, such as the nearest-neighbour distance approach [11], Costes' method [12] and Li's method [13]. However, co-localisation results are still difficult to interpret and compare, and the differentiation of cross-over situations from true co-localisation remains a challenging problem. A study by Landmann [14] considered 3D voxel data and used deconvolution to improve image quality. However the actual co-localisation calculation was performed using existing 2D technique and measured each focal plane separately. Fletcher *et al.* used a robust Monte Carlo randomization based method to measure the statistical significance of co-localisations in 3D [15]. However the use of this method is limited as it made a fundamental assumption that images can be broken into isolated entities and that each foreground object can be isolated from other objects, which depends on the data being punctate in nature. This method also depends on an estimation of the space into which the molecules can be placed.

Robust and accurate co-localisation measurements are needed for a broad range of scientific investigations. In this study, we have developed Co-localisation Intensity Coefficients (CICs) and Co-localisation Binary Coefficients (CBCs) which use rich information embedded in the z-stack of confocal microscopy images and pseudo datasets. This study has produced a novel and unique method to quantify co-localisation by considering the pixel similarities along the z-stack direction.

In this work, we have used an *in vitro* model of central nervous system (CNS) tissue myelination *in vitro* [16] to develop a novel method of accurate and improved co-localisation measurement. In this tissue, cellular processes from oligodendrocytes expressing myelin proteins wrap around axonal processes of neurons (myelination), each of which are fluorescently labelled in the model. However fluorescent myelin signal is also generated from oligodendrocyte cell bodies which can result in false positive co-localisation with axonal signal in multiple planes using traditional measurements; our method reported here minimises such false positives. Additionally two sets of pseudo data were generated to test the robustness of the proposed co-localisation measurements.

## Materials and Methods

### A. Methods

Co-localisation measurements have been used over the last two decades. To quantify the co-localisation between two given colour channels *C_α_* and *C_β_*, most studies take a single focal plane, and compare the pixel values across the two colour channels to identify if overlap occurs. This leads to results with poor accuracy for a number of reasons described below.

#### Overlapping criteria

**Figure 1:**
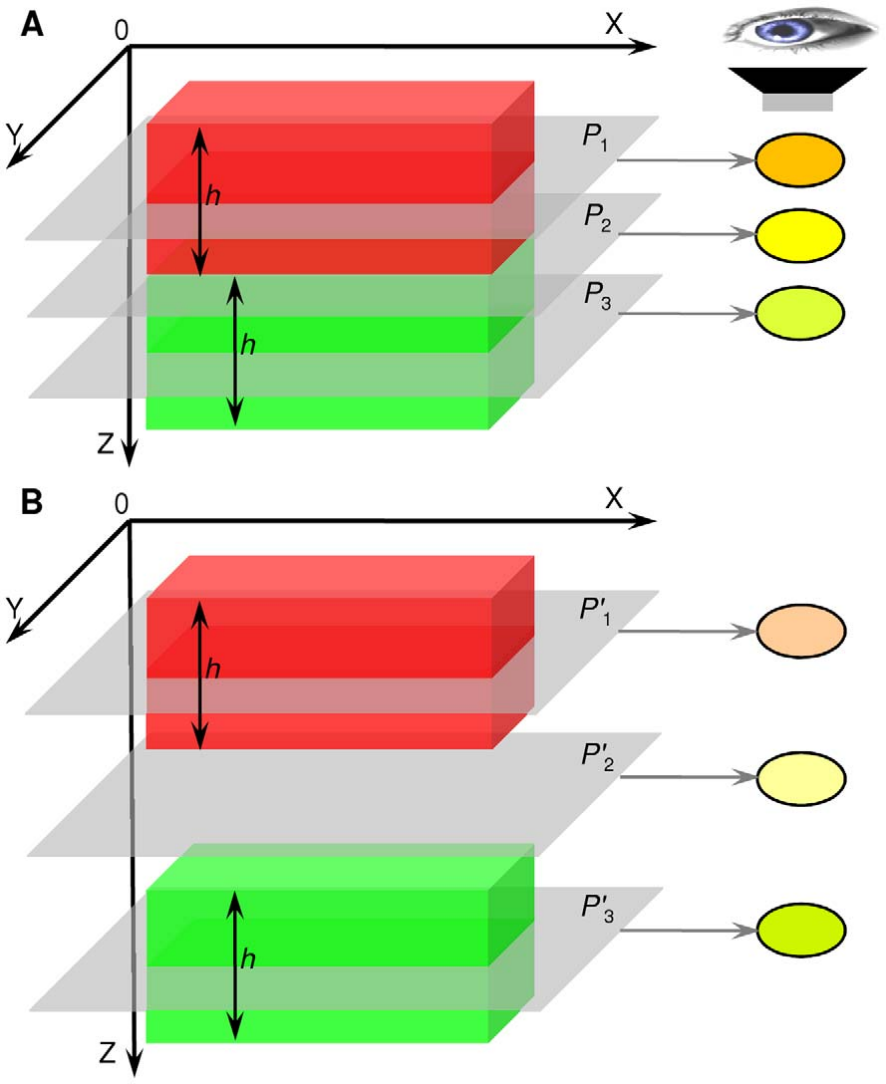
Illustration of generic co-localisation. (A) the red object (top) and green object (bottom) are overlapped and co-localised at focal plane 2, (B) the red (top) object and green object (bottom) are not co-localised, however many co-localisation measurements would incorrectly recognise these as co-localisation.

Well defined criteria regarding how two pixels at a same location across two colour channels are classified as overlapping are lacking. It is insufficient to assume that if two pixels are foreground pixels (through thresholding) that they must be overlapping. A typical example is the so called cross-over situation, which is when foreground objects are on top of each other in the z-stack direction (Figure 1B), rather than actually overlapping (Figure 1A). On a biological level, if given sufficient resolution (e.g. beyond the current maximum confocal microscope magnification), two truly co-localised biological entities appear very close and even touching each other, rather than occupying the same 3D space. In cross-over situations biological entities are actually far from each other, but below the resolution of the instrumentation, they may appear co-localised. Furthermore, spherical aberration, axial under-sampling and the spectral bleed-through from specific signals make the detection of true co-localisation a challenging problem. The majority of current state-of-art co-localisation methods are unable to distinguish between co-localisation and cross-overs, which results in artificially high co-localisation false positivity.

#### Noise

Regardless of its origin, which could be biological or artificial noise can be introduced during sample preparation or at the imaging stage (sensor based) where fluorescence from objects outside the focal plane is visible. This can alter pixel values and may subsequently influence the measurement of co-localisation. Such noise is normally modelled as Gaussian noise, Poisson noise [17] and impulse noise [18]. Therefore, if noise from two colour channels originates from above and below the focal plane independently, they may be considered as co-localised at the converging point in many methods of analysis, e.g. Manders' co-localisation coefficients. However, this noise cannot be ruled out entirely because if the fluorescent signals of both colour channels are originating from the same z direction they must then be counted as co-localised. The method we propose in this study provides the ability to make this discrimination and to recognise co-localisation from noise-polluted signals.

The measurement of co-localisation of overlapping biological entities can be translated into the measurement of similarities between signals in the engineering domain of signal processing, which in the case of digital images is the similarity of image intensities for corresponding pixels.

Observation suggested that given the 3D volumetric model shown in Figure 1A simulating a co-localised situation, the red and green blocks represent small parts of two touching objects, and they all have the thickness (height in the *z* direction) of *h*, where *h* is considerably smaller than the whole thickness of the actual foreground biology entities. Therefore, these two blocks should be recognised as co-localised at any location on focal plane *P*_2_ as the image intensities from both objects at *P*_2_ are similar. When observed under a microscope, focal plane *P*_2_ should appear to be yellow coloured, as shown on the right of Figure 1A. The focal plane which is just above/below the touching plane *P*_2_ (in a small *z* range depending on biological entities to be observed, normally a lot smaller than object heights) should also be considered as co-localised, e.g. focal plane *P*_1_ and *P*_3_. When *P*_1_ and *P*_3_ are observed under a microscope, it may show not necessarily yellow but orange or light green. However, a relative clear indication of a blend of red and green may be observed. This is an important and frequent scenario in which the overlapping of foreground biological entities is not always captured in digital images. Therefore, *P*_2_'s immediate neighbouring focal plane *P*_1_ and *P*_3_ are also regarded as co-localised. However when considering the case in Figure 1B which simulates a cross-over situation, the distance between the red and green blocks is greater (in the *z* direction) than the case shown in Figure 1A (touching). Though the mixing of the red and green colour at focal plane *P*'_1_, *P*'_2_ and *P*'_3_ shows a trace of yellow, especially at *P*'_2_, All these three focal planes (*P*'_1_, *P*'_2_ and *P*'_3_) should all be excluded from co-localisation.

Therefore in this study, to differentiate co-localisation cases (Figure 1A) from exclusion cases (Figure 1B), we developed novel co-localisation measurements CICs and CBCs based on the similarity measurement of z-stack pixel intensity between two colour channels (red and green). This similarity measurement is based on a signal morphological similarity quantification method developed by Lian *et al.* [19].

First of all, all background pixels from all focal planes for both colour channel *C_α_* and *C_β_* are removed. This can be achieved using Otsu's global thresholding method [20]. As this thresholding method can be sensitive to the given image data, therefore for each colour channel, a single threshold value is generated using the Otsu's method which uses all image pixel values across the whole stack of focal planes to define such a threshold value. Therefore, for a given colour channel, all focal planes share a common automatically generated intensity threshold value.

For a non-background pixel with the coordinates of (*x*,*y*) in a random focal plane *P_k_* from colour channel *C_α_*, its pixel intensity is 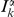. Considering focal plane *P_k_*'s immediate neighbouring focal planes in the z-stack, e.g. [*P_k_*_−2_, *P_k_*_−1_, *P_k_*, *P_k_*_+1_, *P_k_*_+2_], we have a vector of image intensity 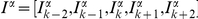. Pixel location (*x*,*y*) at focal plane *P_k_* is only considered to be co-localised if and only if vector *I^α^* is considered to be “similar” to 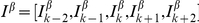 from colour channel *C_β_* By considering a list of neighbouring focal planes in the z-stack, this similarity is defined as a synthesising of each pair of values from vectors *I^α^* and *I^β^*. The entire technical details for this similarity measurement are described in the Information S1 section.

Therefore, we define the Co-localisation Intensity Coefficient for colour channel *C_α_* at focal plane *P_k_* to be:
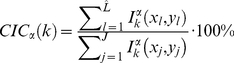
(1)where 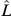 is the total number of foreground pixels in *C_α_*'s focal plane *P_k_* which are considered to be “similar” to its corresponding foreground pixels in *C_β_*, *J* is the total number of foreground pixels in *C_α_* Similarly, we define the Co-localisation Intensity Coefficient for *C_β_* at focal plane *P_k_* to be:
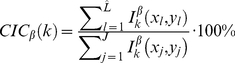
(2)


A previous study [21] suggested measuring the degree of co-localisation using the ratio of the number of co-localised pixels against the total number of positive pixels (greater than a threshold value). For the purpose of a comparison study to this currently accepted method [21], we calculate an alternative measurement, namely Co-localisation Binary Coefficient (CBC).
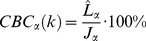
(3)

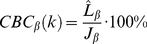
(4)


To measure the overall co-localisation scores for the entire stack of *n* focal planes *P* = [*P_1_*, *P_2_*,…,*P_n_*], the overall CIC and CBC measurements are the average over the z-stack with the exception that if a focal plane does not contain any foreground pixels from both colour channels, its corresponding CIC and CBC measurements from that focal plane are excluded from the calculation of the overall co-localisation scores. Therefore, the overall CICs and CBCs are expressed as:
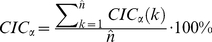
(5)

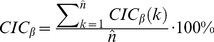
(6)

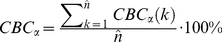
(7)

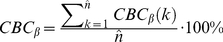
(8)where 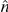 is the number of non-empty frames. The values for eq. (1)–(8) are all in the range of [0%,100%], where 0% indicates no co-localisation and 100% implies full co-localisation.

### B. Materials

To evaluate the robustness of the proposed co-localisation method, we used three sets of test data; z-stack images from murine brain tissue samples and two sets of pseudo volumetric data.

#### *1)* Ethics statement

All animal care and experimental procedures were in accordance with UK Home Office guidelines (Certificate of Designation 5012, PPL 2675) and approved by the Queen's University Belfast Ethical Review Committee.

#### *2) Organotypic CNS Slice Cultures*

Images of murine brain tissue samples were obtained from a programme of research focused on CNS demyelinating diseases. Briefly, CNS Organotypic slice cultures (OSC) were experimentally demyelinated and allowed to remyelinate in vitro.

Mice were bred in accordance with UK Home Office guidelines. OSC were prepared according to a modified protocol of the published method of Stoppini et al. [22]. Briefly, the cerebral cortex was obtained from 6 to 10 day old pups and 300 µm saggital slices were taken using a vibroslice (Camden Instruments). OSC were cultured for 12 to 16 days at 37°C in a humidified atmosphere with 5% CO_2_.

OSC were fixed in 4% paraformaldehyde (Sigma) in phosphate-buffered saline (PBS) for 2 hours (RT) and permeabilised with 1% Triton X-100 (Sigma) in PBS for 40 mins (RT). Slices were then blocked with 10% normal goat serum (Invitrogen) in 0.2% Triton X-100 in PBS for 1 hour (RT). Primary and secondary antibodies were diluted in 1% goat serum, 0.2% Triton X-100 in PBS, slices were incubated in primary antibody for 2–3 day and secondary antibody overnight (both at 4°C). Antibodies used were, anti-MBP (1∶200; Myelin Basic Protein, rat monoclonal, Millipore), anti-Neurofilament 200 (1∶600; mouse monoclonal RT97, Millipore). Goat anti-mouse and goat anti-rat antibodies conjugated with Alexa Fluor-488 and Alexa Fluor-594 (1∶200; all from Invitrogen) were used as secondary antibodies as indicated. Slices were then mounted on a glass microscope slide with Prolong Gold (Invitrogen, UK; 1.47 RI), covered with a 0.17 mm thick glass coverslip and sealed with black nail polish. Slides were covered in aluminium foil and kept at 4°C until ready for use. Imaging of immuno-stained OSC was performed using a laser-scanning confocal microscope (Leica TCS SP5, UK) between 1 and 5 days after staining.

Excitation of fluorescently labelled secondary antibodies by the respective excitation lasers (488 nm and 594 nm) was sequentially scanned. Image stacks of between 5 and 10 µm in depth were acquired at 0.5 µm intervals using a 40× oil immersion objective (Numerical Aperture = 1.25) with the pinhole diameter set to the equivalent of 1 Airy unit. Intensity of fluorescence was adjusted using the smart gain to ensure brightest objects were maximal without being saturated and background was corrected using the smart offset feature of the LAS AF software, these adjustments were made for a control slice and remained constant for imaging all slices from that experimental replicate. Images were captured at a scanning frequency of 400 Hz with no correction for aberrations at a resolution of 1024×1024 pixels (0.25 µm/pixel). For analysis, image stacks for each colour channel were converted to AVI files with lossless compression.

#### *3) Pseudo Volumetric Data*

To validate readouts from biological images, we generated a wide range of co-localisation pseudo data to incorporate total co-localisation (100%) through a range of partial co-localisation, down to a complete absence of co-localisation (0%). Using this large set of artificially generated data we evaluated CIC and CBC co-localisation measurements.

The proposed method in this study uses z-stack information for the measurement of overlapping colour channels. Therefore, single 2D images are not sufficient. To simulate the physical zooming process of confocal microscopy, a fifteen-frame 3D volumetric dataset was generated using the following method with the assumption that true in-focus objects are located between frame number 7 and 8.

A single colour channel image I was created with the size of 256×256 pixels and first painted in black. Sixteen white dots, to simulate foreground objects, were then drawn on the image with the diameter of 19 pixels, and scattered in the image. Additional zero-mean Gaussian white noise with the variance of 0.01 was then added to the image. This image was then normalised to the intensity range of [0,1].

Movement of the microscope lens up and down changed foreground objects from blurred to sharp, then to blurred again. This process was modelled as a Gaussian function in this study.

To simulate a degree of blurriness, image I was filtered using the following Gaussian lowpass filter:
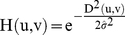
(9)where 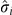 is the standard deviation of the Gaussian lowpass filter. Its value is given by
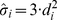
(10)D(u,v) calculates the meshgrid frequency matrix [23] with the size of *M*×*M*, where *M* is the edge length of the squared Gaussian lowpass filter (in pixels)
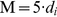
(11)where *d_i_* is the frame distance, which defines the distance between the current blurred image to the in-focus (shape) view of the foreground object. Without knowing the foreground object dimensions and distances among focal planes in µm, we define the value of *d_i_* for the top to bottom (i = 1,2,…,15) using the following lookup table (in units):
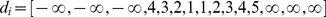
(12)


**Figure 2:**
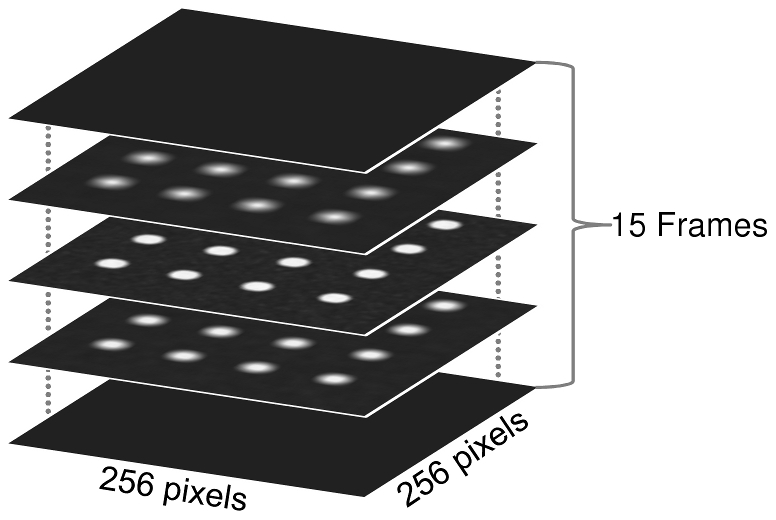
Illustration of the template stack consisting of 15 frames 256×256 pixel images. The template focal plane I is plane number 8, which is in the middle of all focal planes.

When *d_i_* = −∞ or *d_i_* = ∞, it represents when the foreground object is so out-of-focus, that it becomes invisible (black). Therefore, the top three and bottom three frames are completely black. In such a way, we generated a stack of fifteen 256×256 pixels frames (shown in Figure 2) for analysis and named this stack of images 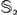.

To test co-localisation, a single stack 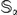 for colour channel *C_α_* is not enough, thus, another stack 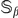 is needed to represent colour channel *C_β_*, where colour channel *C_β_* can be generated using 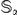.

**Figure 3:**
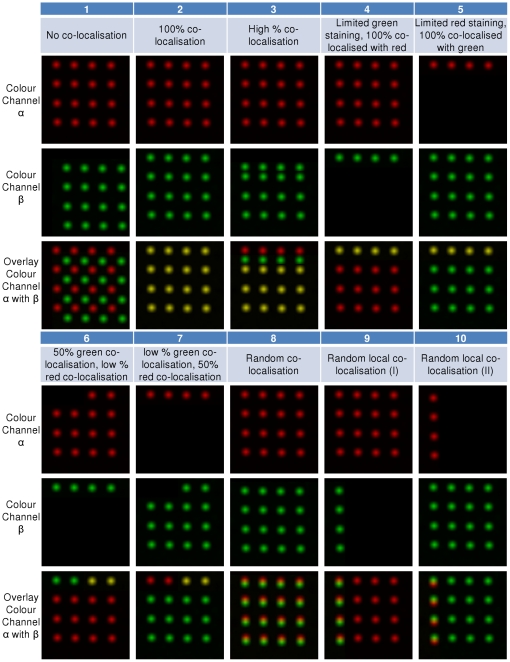
Ten pseudo-test cases from 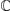
 **.** Only the 5th frames from the top of each stack (15 frames in total) images are shown for illustration purposes. Each image has the size of 256×256 pixels.

Initially, a 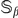 is created by replicating each frame in 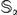. The *xy*-location of each dot in 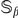 is then re-arranged. Of note, all dots lying in one cylinder across all frames in 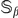 are re-arranged in the same way. A set of ten stacks 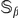 were used to simulate 10 various co-localisation situations. We then superimposed the stack 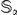 with red colour, and all stacks 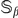 with green colour, and examples of these 10 test cases 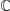 are shown in Figure 3.

To simulate the situation when two foreground objects appear overlapping due to close proximity but actually are spatially separated in the z-stack direction (cross-over situation), rather than truly co-localising, we proposed the following set of test cases 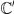.

Given 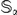, the top three black frames are first removed, an extra seven all black frames are then appended to the bottom of the set, giving the total of nineteen frames and this stack was named 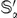. The first nine frames in 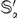 contains foreground objects, whereas the last nine frames are only black images.

**Figure 4:**
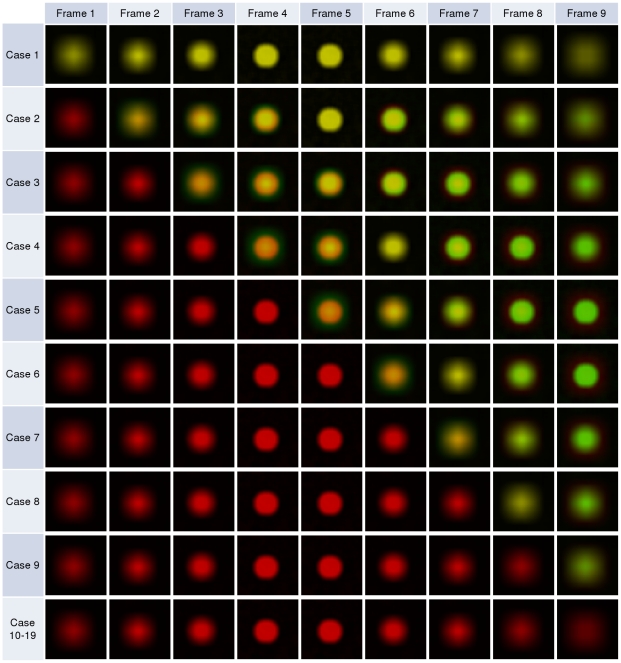
Illustration test cases from 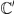
 **.** Only the overlaid red-green colour images are shown here. Each row represents the first nine frames of a test set. As all 16 dots in a frame are identical, to save space, only one dot (20×20 pixels) is shown. For test case 10 to test case 19, the first nine frames are the same.

To generate a co-localisation test stack 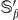 for 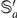, 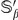 first replicates all frames from 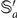. A number of *n'* black 256×256 pixel images are then inserted to the top of 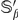, and the bottom *n'* frame from 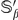 is then removed, where 0≤*n'*≤18. This resulted in another 19 test cases in 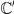. An illustration of 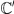 is shown in Figure 4.

## Results

### A. De-/Re-myelination Data

**Figure 5:**
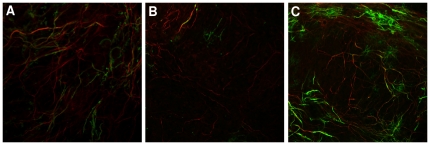
Three examples of murine organotypic brain slice cultures. (A) Control OSC, (B) Demyelination, (C) Remyelination. *All these figures are single focal planes taken from a set of 5 control, 5 demyelination and 7 remyelination stacks. MBP (Myelin) is shown in green and N200 (Axons) is in red.

The proposed CICs and CBCs measurements were first evaluated within a large on-going project investigating remyelination in murine organotypic brain slice cultures. The purpose of quantifying co-localisation of fluorescent signals in these images is to quantify myelination and remyelination, both of which are key biological processes in health and disease. A hallmark of (re)myelination is true co-localisation of myelin with axons and quantification can be distorted by additional myelin signal from myelin-forming cells (oligodendrocytes) in distant planes. Examples of images of control, de-/re-myelinating slice cultures are shown in Figure 5. At the demyelination phase, a reduction of co-localisation is anticipated to be a result of myelin loss from the axons. During the remyelination process (repair phase),oligodendrocytes are present at much greater numbers and producing more MBP signal. However, the presence of these cells does not imply true remyelination as myelin sheaths from these cells must both engage and wrap around axons in order to carry out actual remyelination Based on the biological basis of this model, which can require up to 10 days to remyelinate, we anticipate a lower proportion of myelinated axons in repairing slices (3 days post demyelination) than in control healthy slices. This biological knowledge can be used as a guide to judge the correctness co-localisation measurements, whereas the results from pseudo datasets can also be used to confirm this statistically in the following section.

**Figure 6:**
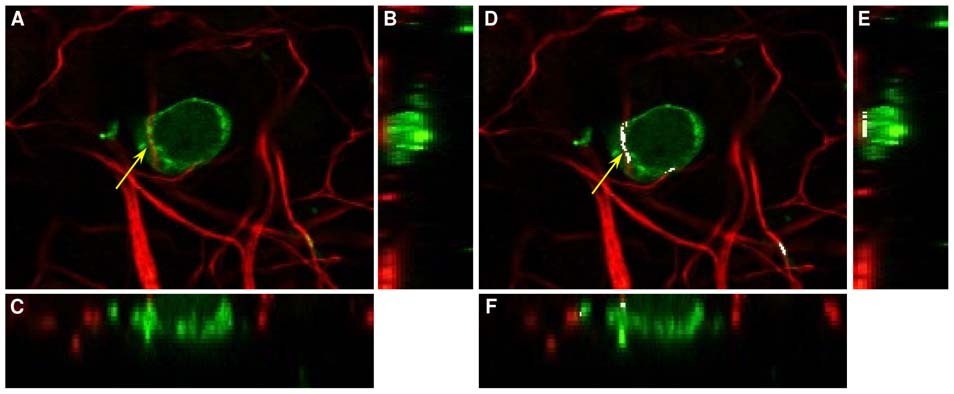
Comparison of *CIC_α_* measurement and Manders' 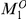
 **using examples from murine organotypic brain slice cultures.** (A) An example of a focal plane from murine OSC at 40× magnification, showing *CIC_α_* = 0% using our method, (B) side view of the z-stack (view in the *x* and *z* plane) for Figure A, (C) view in the *y* and *z* plane for Figure A, (D) The same example image as Figure A showing the result using Manders' method, where the white region indicates co-localisation, (E) side view of the z-stack for Figure D, (F) bottom view of the z-stack for Figure D.

As part of the study, we sought to quantify the amount of co-localisation between axons and myelin in image stacks of control (*n* = 5), *in vitro* demyelinated (*n* = 5) and remyelinated samples (*n* = 7). During this process, oligodendrocyte cell bodies and extending processes both express MBP which increases the likelihood of potential overlap error due to the MBP (green) positive cell bodies occupying considerable space amongst N200 (Red) positive axons. This is exemplified in Figure 6 where a stained axon (red) aligns under an oligodendrocyte cell body (green).

For the quantification of the extent of myelination, the most appropriate measurement is the proportion of red co-localised with green. Therefore, we used the proposed *CBC_α_* and *CIC_α_*. Rather than only referencing other comparison studies in the literature which evaluate the performance of popular co-localisation studies, or trying to prove popular co-localisation measurements are not sufficient in a mathematical/theoretical manner, we performed a direct comparison of our proposed method against the following list of 7 popular co-localisation measurements from the literature. They are

- *PC*: Pearson's coefficient [24]
- *PC^C^*: Pearson's coefficient using Costes' automatic thresholding method [12]
- *CCF*: the maximum cross-correlation coefficient [25]
- *k*_1_: overlap coefficient [5]
- *M*_1_: Manders' co-localisation coefficient [26]

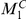
: Manders' co-localisation coefficient with Costes' automatic thresholding method [12]

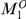
: Manders' co-localisation coefficient with Otsu automatic thresholding method [20] (same as in our proposed method).

**Figure 7:**
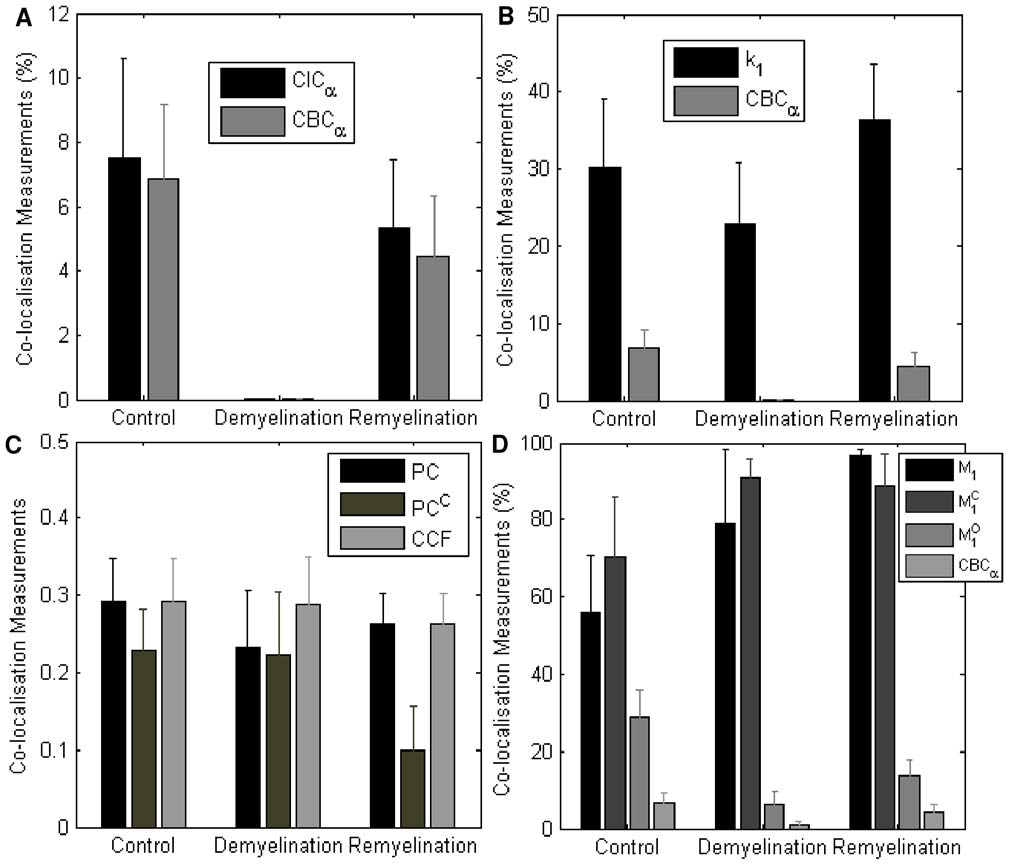
Performance evaluations of *CBC_α_* and *CIC_α_* with other 7 popular co-localisation measurements from murine organotypic brain slice cultures. Co-localisation measurements are evaluated for control, demyelinated and remyelinated samples. Error bars represent SEM. (A) Performance comparison between *CBC_α_* and *CIC_α_*, (B) Performance comparison between *CBC_α_* and overlap coefficient *k*_1_, (C) Performance comparison among Pearson's Coefficient (*PC*), Pearson's coefficient using Costes' automatic thresholding (*PC^C^*) and the maximum cross-correlation coefficient (*CCF*), (D) Performance comparison among *CBC_α_*, Manders' co-localisation coefficient (*M*_1_), Manders' co-localisation coefficient with Costes' automatic thresholding (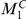) and Manders' co-localisation coefficient with Otsu automatic thresholding (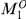). *The measurements presented in Figure A, B and D multiplied by 100% to be comparable with *CBC_α_*.

When comparing the results obtained from *CBC_α_* and *CIC_α_* (Figure 7A), results are very almost identical, with the values of *CBC_α_* being slightly smaller than *CIC_α_* (*p* = 0.7626). These results suggest the amount co-localisation reduces to be almost 0 (mean *CBC_α_* = 1.10%, and mean *CIC_α_* = 1.44%) at the demyelination stage, and after remyelination, a degree of co-localisation (*CBC_α_*: 65.25% and *CIC_α_*: 70.82%) is re-established however smaller than the co-localisation readings from the control group, as would be expected biologically. Both *CBC_α_* and *CIC_α_* readings are expected from each phase of our OSC model. At the demyelination stage, the majority of myelin has been destroyed; therefore an absence or major reduction of co-localisation of myelin with axons is expected. At the remyelination phase, we would expect increasing percentages of myelination (co-localisation) to be observed, but lower than the healthy controls that are fully myelinated as discussed above.

We compared the popular overlap coefficient *k*_1_ with *CBC_α_*, results are shown in Figure 7B. Statistical test shows that the co-localisation scores between the two measurements for all the samples are significantly different (*p* = 1.9008×10^−5^). As can be seen, the *k*_1_ reading at the demyelination stage is overly high (mean *k*_1_ = 0.2280), though or little co-localisation is in fact expected (e.g. Figure 5B). It also shows that the *k*_1_ score at remyelination (mean *k*_1_ = 0.3639) is higher than the control group (mean *k*_1_ = 0.3030) which also contraindicates the biological properties of remyelinating slices.

As the value for *PC*, *PC^C^* and *CCF* are all in the range of [−1, 1], these three measurements are plotted and compared together in Figure 7C. Observation shows the co-localisation readings between control and demyelination are similar. Subsequent statistical analysis of differences (t-test) using control and demyelination data, which biologically should have significantly different co-localisation measurements (see Figure 5A, B), suggests these three measurements are not ideal to exhibit the difference between these two groups (*p_PC_* = 0.5430, 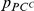 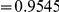 and *p_CCF_* = 0.4902, comparing to *p_CBC_* = 0.0432).

Finally, we tested the performance of *CBC_α_* against *M*_1_, 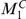 and 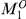. Subsequent results from these tests are shown in Figure 7D. It is clear that co-localisation measurements from *M*_1_ and 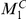 are inaccurate, as i) their co-localisation scores at demyelination are too high (>70%), and their remyelination scores are higher than controls. Results from 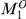 (mean 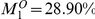 for control, 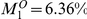 for demyelination and 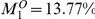 for remyelination) are better however still considerably higher than *CBC_α_* readings (mean *CBC_α_* = 6.85% for control, *CBC_α_* = 1.10% for demyelination and *CBC_α_* = 4.47 for remyelination). Statistical analysis of combined control and de-/re-myelination samples shows that the co-localisation scores between *CBC_α_* and 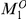 are significantly different (*p* = 0.0036). A large number of cross-over situations, which are the random co-localisation of OL cell bodies with axons and crossing OL myelin processes that co-localise at discrete points of axons rather than longitudinally, still exist using 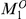, whereas our *CBC_α_* scores significantly reduced these types of false positives. A good example is shown in Figure 6 where a typical cross-over between a cell body (green) and axon is excluded using our method, however, this was incorrectly classified as co-localisation using Manders' method. It is most evident at the demyelination stage with the average *CBC_α_* = 1.10%. Furthermore, 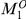 measurements also result in high standard deviation, e.g. at the demyelination stage with 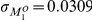, comparing with 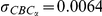.

Based on the biological properties of the OSC model during de-/re-myelination, this comparison study suggests that the described method in this study correctly identifies levels of co-localisation whilst also improving sensitivity by ruling out false positives, which are largely cross-over situations.

### B. Co-localisation/Cross-Over Test Using Test Set 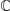


The 10 pseudo volumetric test cases from set 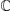 were also tested with multiple measurements. Results for all test cases from 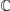 (Figure 3) are shown in Table 1. Visual scoring results are also provided in this table to serve as ground truth for performance evaluation. Due to the way these pseudo data are designed, the degree of overlapping and co-localisation for test cases 1–7 in Figure 3 can be precisely determined. To allow a safe margin for error, the visual scores for test case 8–10 are given as a small range.

**Table 1:** List of evaluation results for the 10 test cases from set 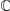.

Test cases	α Visual results	*M(1)*	CIC(α)	CBC(α)	σ(CIC(α))	σ(CBC(α))	β Visual results	*M(2)*	CIC(β)	CBC(β)	σ(CIC(β))	σ(CBC(β))
1	0	0	0	0	0	0	0	0	0	0	0	0
2	100	100	100.00	100.00	0.00	0.00	100	100	100.00	100.00	0.00	0.00
3	75	74.9	75.01	75.04	0.00	0.00	75	74.9	75.01	75.04	0.00	0.00
4	25	25	24.99	24.96	0.00	0.00	100	96.6	96.73	94.74	0.03	0.03
5	100	96.6	96.73	94.74	0.03	0.03	25	25	24.99	24.96	0.00	0.00
6	14.29	14.2	14.26	14.25	0.00	0.00	50	48.3	48.29	47.31	0.01	0.02
7	50	48.3	48.29	47.31	0.01	0.02	14.29	14.2	14.26	14.25	0.00	0.00
8	25–30	**43.9**	29.55	26.78	0.03	0.04	25–30	**44**	27.45	26.78	0.04	0.04
9	5–10	**11.4**	7.61	6.94	0.01	0.01	25–30	**44.1**	27.13	26.20	0.04	0.04
10	25–30	**44.1**	29.46	26.09	0.03	0.03	5–10	**11.4**	6.99	6.91	0.01	0.01

σ(•) is the standard deviation for either CIC or CBC for all the focal planes in one stack of test case. *M(1)* refers to Manders' 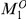 and *M(2)* refers to Manders' 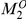, they are multiplied by 100% to be comparable with our results.

Results from Figure 3 indicate that the proposed CIC and CBC scores are similar to the visual scoring results from an experienced neuroscientist. Visual inspection also suggests that the influence of noise towards CICs and CBCs is minor.

Comparing with Manders' co-localisation coefficients 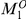 and 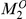 using a same set of background threshold values from Otsu's method as in our proposed method, our results are very similar to 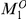 and 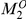 (column 3 and 9 in Table 1).

**Figure 8:**
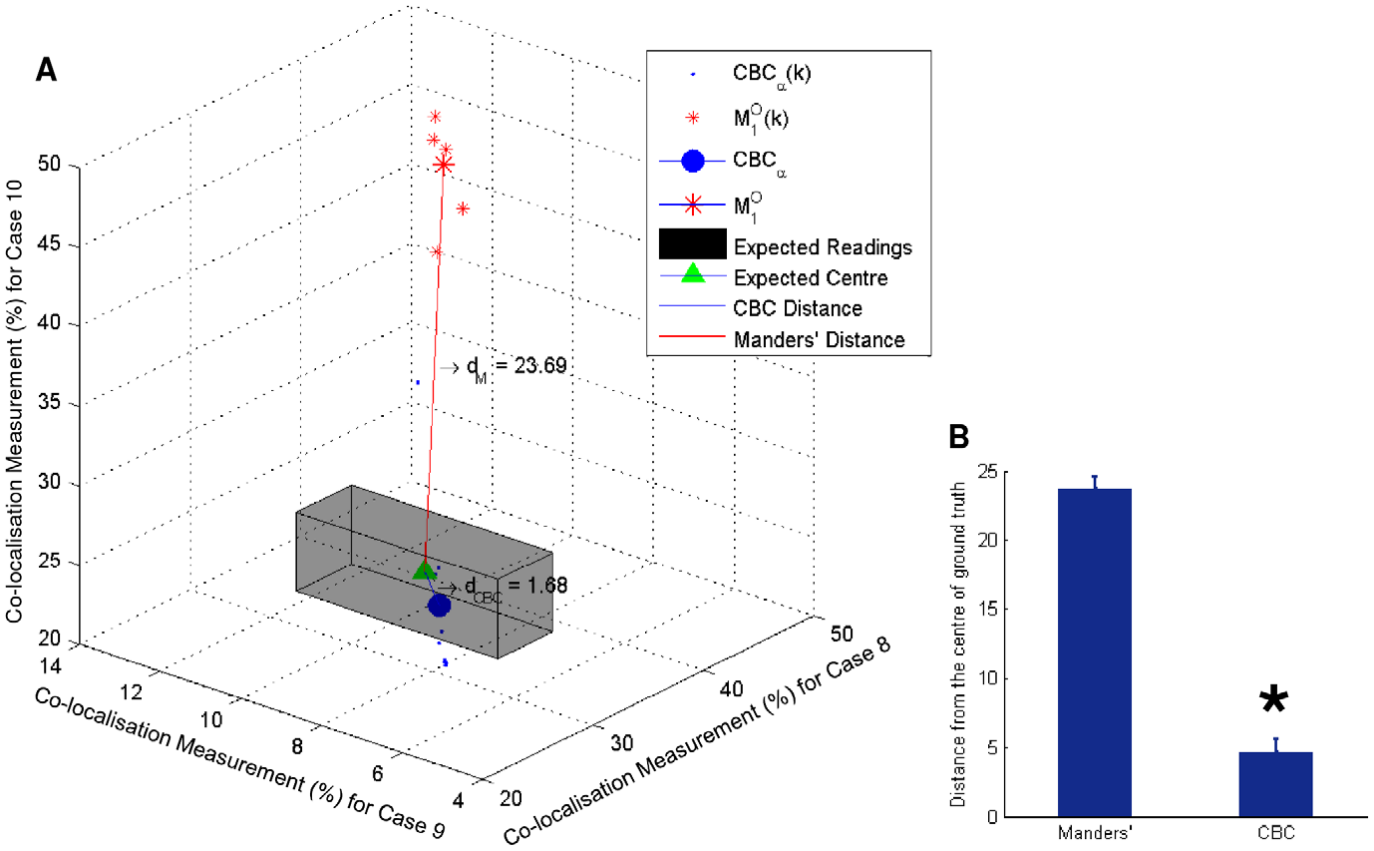
Performance comparison between *CBC_α_* and Manders' method shows that our co-localisation measurement is a lot closer to the ground truth value than measurements obtained using Manders' method with Otsu's thresholding method. (A) Co-localisation measurements of *CBC_α_*(*k*) (blue dots) and Manders' 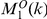 (red star) for all non-empty frames in test cases 8–10 is plotted. The range of ground truth is shown in the gray box with its centre *V_t_* shown in a green triangle. The *CBC_α_* value, which is also the geometric centre of *CBC_α_*(*k*), is shown by the large blue dot, and the value of 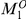, which is the geometric centre of 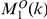, is shown by the large red star. Results show that the distance between 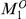 and *V_t_*, *d_M_* = 23.06 is considerably larger than the distance between *CBC_α_* and *V_t_*, *d_CBC_* = 1.68, which suggests greater accuracy of our method in comparison with Manders' method, (B) two sample t-test results show that the individual distances *d_CBC_*_(*k*)_ (between *CBC_α_*(*k*) and *V_t_*) are significantly smaller than the distances *d_M_*_(*k*)_ (between 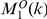 and *V_t_*). *The y-axis in Figure B indicates *d_CBC_*_(*k*)_ and *d_M_*_(*k*)_, which is measured using the percentage of co-localisation.

**Figure 9:**
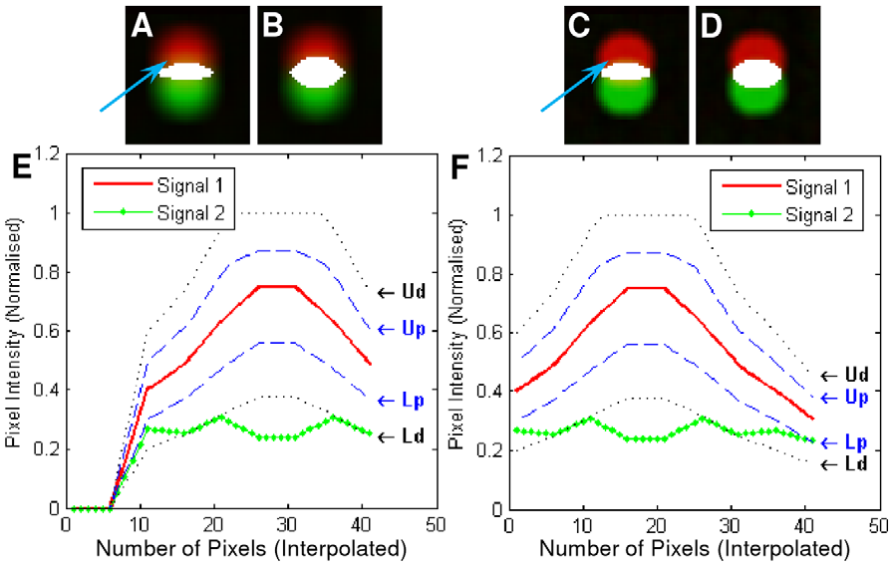
Illustration of identifying cross-over situations using our proposed method in comparison with Manders' co-localisation coefficient masks. The overlaid masks are shown in Figure A–D, where the white regions are the co-localised regions. (A) Results using our method from frame 6 of test case 8 in 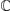, (B) Manders' co-localisation coefficient mask using frame 6 of test case 8 in 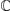, (C) Results using our method from frame 8 of test case 8 in 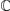, (D) Manders' co-localisation coefficient mask using frame 8 of test case 8 in 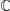, (E) the plot of image intensity at a pixel pointed by the blur arrow in Figure A using a nine frame neighbourhood using our method, (F) the plot of image intensity at a pixel pointed by the blur arrow in Figure C using a nine frame neighbourhood using our method. Figure A and C represent different degree of blur.

For test cases 1–7, our CICs and CBCs measurements are very similar to 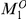 and 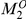, however for test cases 8–10, both CICs and CBCs are considerably smaller than 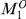 and 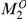. If we plot both *CBC_α_*(*k*) and 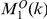 results (for all the non-empty frames) for cases 8–10 together, as illustrated in Figure 8A, results show that our *CBC_α_*(*k*) are largely scattered around the expected ground truth region (the gray box), whereas the 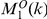 readings are far from it. The geometric centre of *CBC_α_*(*k*), which is the *CBC_α_* for the 3 test cases 8–10, is illustrated with a large blue dot, where as the geometric centre of 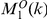, which is the 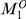 for the 3 test cases 8–10, is illustrated with a large red star, Result shows that the Euclidian distance between the expected ground truth (the green triangle *V_t_*) and *CBC_α_*, *d_CBC_* = 1.68, is considerably smaller than the distance between *V_t_* and 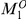, *d_M_* = 23.69. The values of *d_CBC_* and *d_M_* are measured using the percentage of co-localisation. We verified this finding statistically using a two sample t-test for all the individual distances *d_M_*_(*k*)_ (between 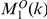 and *V_t_*), and *d_CBC_*_(*k*)_ (between *CBC_α_*(*k*) and *V_t_*). Statistical test results in Figure 8B demonstrate that *d_CBC_*_(*k*)_ is significantly smaller than *d_M_*_(*k*)_ (*p* = 6.27×10^−11^). This provides a clear indication that our co-localisation measurement method produces results significantly closer to the ground truth, and hence has greater accuracy than the Manders' measurements. This finding can be both explained and enhanced by the fact that our method is able to identify and exclude a large quantity of cross-overs, as shown in Figure 9, which significantly reduces false positive co-localisation results.

It is also noticeable the standard deviations for CICs and CBCs for all focal planes are minor σ≤0.04. Given that the major differences among focal planes are the amount of blurriness artefacts, it appears the proposed method is robust in regard to blurriness.

**Figure 10:**
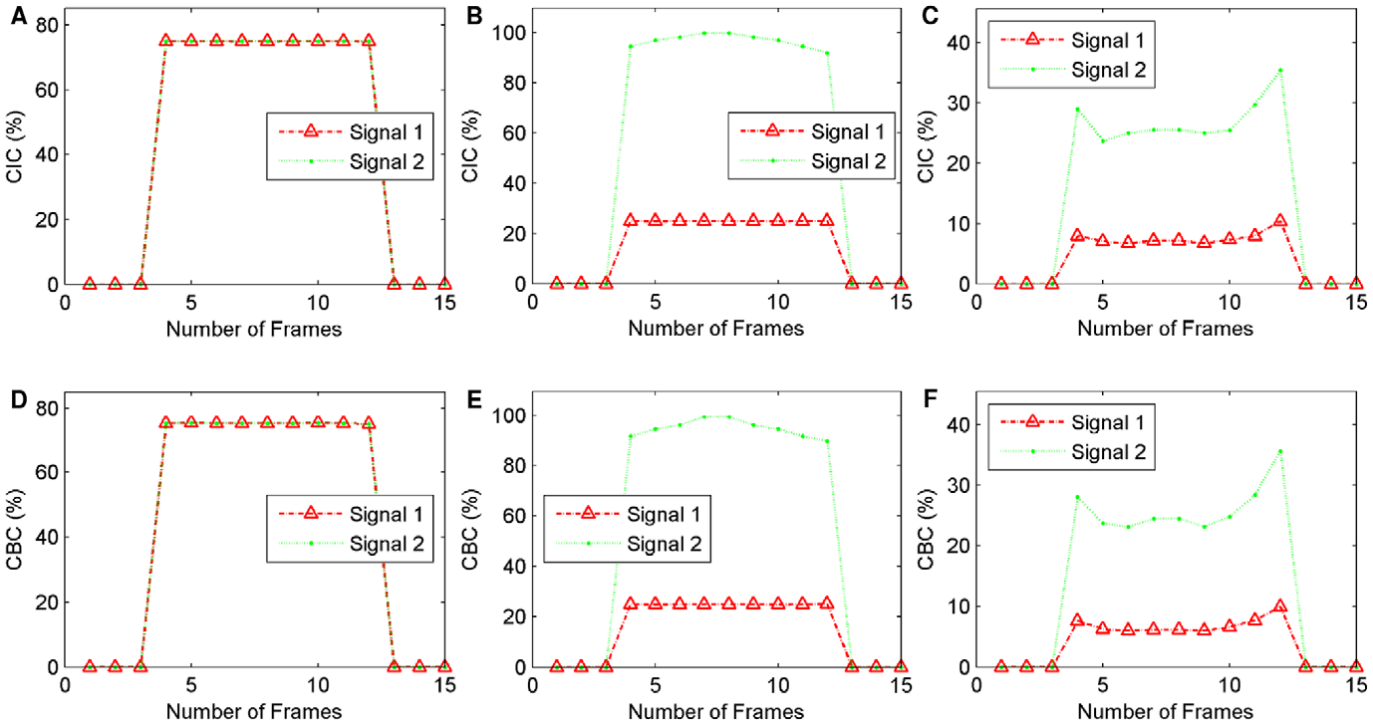
Individual frame CIC and CBC scores for three test cases from 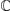
 **.** (A) *CIC_α_*(*k*) and *CIC_β_*(*k*) for all the frames *k* = 1,2,…,15 from test case 3, (B) *CIC_α_*(*k*) and *CIC_β_*(*k*) for all 15 frames from test case 4, (C) *CIC_α_*(*k*) and *CIC_β_*(*k*) for all 15 frames from test case 9, (D) *CBC_α_*(*k*) and *CBC_β_*(*k*) for all 15 frames from test case 3, (E) *CBC_α_*(*k*) and *CBC_β_*(*k*) for all 15 frames from test case 4, (F) *CBC_α_*(*k*) and *CBC_β_*(*k*) for all 15 frames from test case 9.

Three examples of individual frame CIC and CBC scores are plotted in Figure 10.

Plots in Figure 10B and E show the results from test case 4. The *CIC_α_*(*k*) and *CBC_α_*(*k*) for all the frames (*k* = 1,2,…,15) are quite stable across all frames, as indicated as Signal 1 (red) in the plots. The plots for *CIC_α_*(*k*) and *CBC_α_*(*k*) (Signal 2 green) show their values are peaked at frame 7–8 and decrease towards the two sides until frame 4 and 13. This can be explained by the fact that when Otsu's global thresholding method was used to identify foreground objects, more blurred pixels from colour channel *C_β_* (green) were kept as foreground objects (compared with colour channel *C_α_*). This was expected as *C_β_* contains significantly less foreground objects compared with *C_α_*. Results can be improved using a number of pre-processing techniques, such as image restoration [7], and blur detection [27], [28] however the pre-processing of images is beyond the scope of this current study.

Plots in Figure 10C and F show the results from test case 9. The two peaks at frame 4 and 13 are expected and can be explained by the fact that these frames from both colour channel *C_α_* and *C_β_* are largely blurred and a significant amount of blur is recognised as overlapping and co-localising.

### *C. *Co-localisation/Cross-Over Test Using Test Set 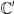


The test results for the 19 test cases in 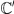 are listed in Table 2. This set is testing if the proposed method can recognise and differentiate co-localised foreground objects from situations when foreground objects are cross-over.

**Table 2:** List of evaluation results for the 19 test cases from set 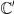.

Test cases	*M(1)*	CIC(α)	CBC(α)	*M(2)*	CIC(β)	CBC(β)
1	100.00	100.00	100.00	100.00	100.00	100.00
2	89.0	95.57	93.43	90.6	94.60	92.16
3	78.0	31.48	35.18	81.4	30.83	34.69
4	65.5	8.85	14.06	70.7	8.36	13.97
5	51.9	6.75	10.46	59.0	6.19	10.47
6	39.4	0.00	0.00	46.3	0.00	0.00
7	26.4	0.00	0.00	31.5	0.00	0.00
8	15.9	0.00	0.00	19.3	0.00	0.00
9	6.8	0.00	0.00	8.4	0.00	0.00
10–19	0.00	0.00	0.00	0.00	0.00	0.00

*M(1)* refers to Manders' 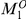 and *M(2)* refers to Manders' 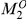, they are multiplied by 100% to be comparable with our results.

Using our method, as the test cases move down, the percentage of co-localisation regions decreases rapidly to 0% at test case 6 (Table 2) where the two foreground objects are no longer touching each other. This phenomenon of decreases in co-localisation complies with how our artificial data were generated. These results were then verified by visual inspection by an independent expert blinded to the results, which confirmed the accuracy of these results.

**Figure 11:**
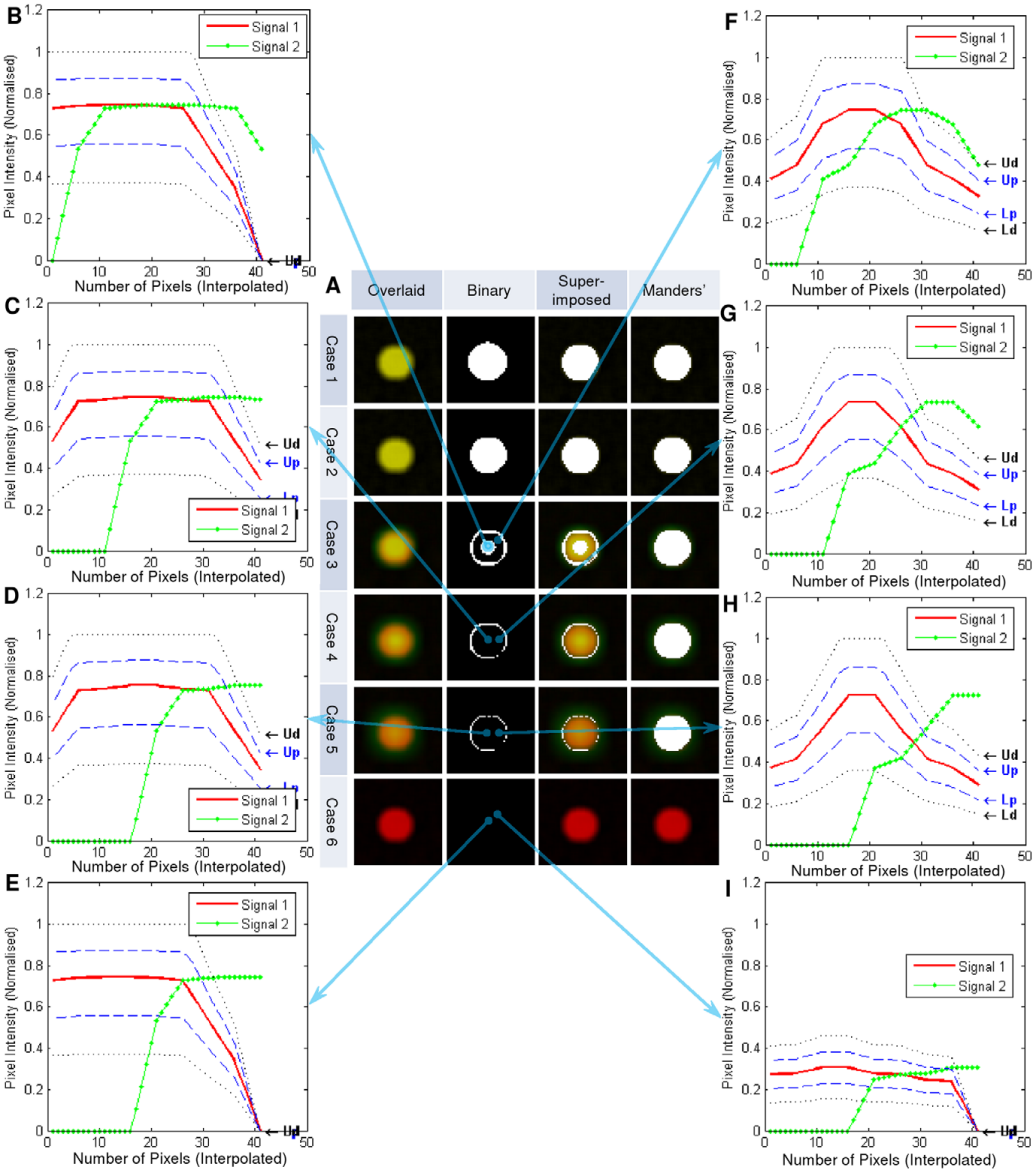
Co-localisation test results using frame 5 from test cases 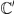
 **as an examples.** As all dots per focal plane represent the same results, only one dot is shown here in Figure A. (A) Test results using our method in comparison with Manders' co-localisation coefficient overlaid masks. (B)–(I) pixel similarity plots at the pixel indicated with arrows. *In Figure A, column ‘Overlaid’ indicates the unprocessed overlaid colour channel *C_α_* and *C_β_*. Column ‘Binary’ shows the binary mask where white indicates the region of co-localisation. Column ‘Super-imposed’ shows the superimposition of column ‘Binary’ on top of column ‘Overlaid’, whereas column ‘Manders'’ is the overlaid mask from Manders' co-localisation coefficient measurements. In Figure (B)–(I), Figure B represents a good co-localised case, whereas Figure (C)–(H) are all not co-localised because they do not satisfy the *ASCI* similarity measurements, whereas Figure (I) is not co-localised because the image intensity values for both colour channels are below the Otsu's threshold. Test cases 7–19 are not shown here as their binary masks are completely black using our method as in case 6, indicating 0% co-localisation.

To use frame 5 as an example, good co-localisation is shown in Figure 11B. Figure 11C–H showed the similarity measurement plots of the six pixels indicated with arrows. These pixels are classified as exclusion simply because they represent cross-over situations, though their pixel intensity values from colour channel *C_α_* and *C_β_* are similar at the pixel. However, when considering its 9 frame neighbourhood, they have been correctly recognised as cross-over and classified as exclusion. Figure 11I is also classified as not co-localised because the image intensity values at that pixel for both colour channels are below the Otsu's threshold and therefore they are recognised as background.

Figure 11B–E represent the centre of the dots, whereas Figure 11F–I show the edge area of the dots. Though their intensities are not the same in these eight cases, the proposed method recognised and correctly classified all of these cases. In comparison, the last column of Figure 11A showed the results using Manders' co-localisation coefficients with Otsu thresholding as our proposed method, and each test case (case 1–5) has a large and similar overlaid mask indicating its incapability of recognising cross-over.

**Figure 12:**
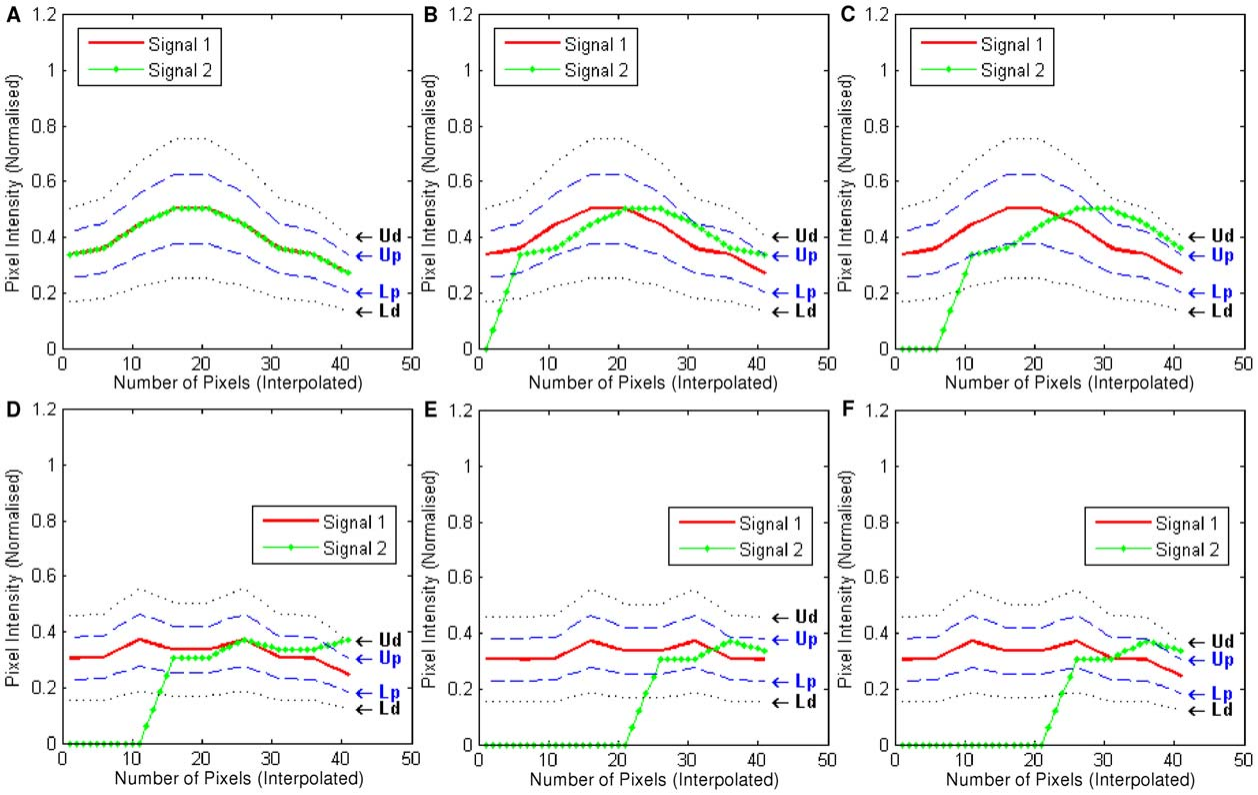
Plots of an edge and centre point of a dot from frame 5 for the first six test cases in 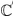
 **.** Signal 1 is the image intensity values for a nine frame neighbourhood from colour channel *C_α_*, whereas Signal 2 stands for *C_β_*. For both Signal 1 and 2, linear interpolation is applied to generate more data points.

In these examples the regions which are more likely to be co-localised are either the centre of the dot, or the blurred edge regions (Figure 11). The identification the outside edge of these objects is due to the smooth nature of image intensities across the neighbourhood of nine frames, as illustrated in Figure 12. The interpolated image intensity signal (Signal 1) for *C_α_* is smooth, without sharp changes. Therefore, it matches with Signal 2 at a nine-frame neighbourhood which was expected by the authors. These specific co-localised edge pixels could be either regarded as blur, which may or may not be inside the true boundary of the foreground object, or as another object of weak fluorescent intensity. If it is blur artefact, it can be reduced or even removed by image restoration and digital noise signal reduction techniques in either pre-processing or post-processing steps. However, if it is a weakly stained biological entity, our analysis certainly recognised the correct co-localisation region. Similarly, the centre part of the dot also has smooth image intensities for both colour channel *C_α_* and *C_β_*. Therefore, they are also considered as co-localised.

## Discussion

This study presents both a novel and more reliable method for the quantification of co-localisation which we have utilised in neurobiology but could potentially facilitates a vast range of other biological studies. The performance of this co-localisation measurement is evaluated using OSC and two sets of pseudo data, and superior performance has been demonstrated to be statistically significant. In the comparison study with 7 popular 2D co-localisation measurements, readings (CICs and CBCs) obtained from our method are significantly more accurate than others. This highlights the main strength of our proposed method, its capability of identifying and ruling out a significant amount of cross-over situations and greatly reducing false positives for co-localisation measurements. In comparison with recent studies which have utilised 3D voxel data for co-localisation measurements, our method is simple and computationally inexpensive as we used a simple 1D signal similarity measurement without the need for complex 3D volumetric modelling.

The co-localisation measurement from this study measures the image intensity similarities along the z-stack direction, and it is robust in recognising and excluding a significant amount of cross-over. As the values of CICs and CBCs are in the range of [0%,100%], results are easy to interpret and analyse.

The similarity measurement in this study is reported to be robust against noise (Poisson, Gaussian or impulse noise) [19]. The proposed Co-localisation Intensity Coefficients (CICs) and Co-localisation Binary Coefficients (CBCs) made use of rich z-stack information for the measurement of co-localisation. It is proven to be robust in identifying and removing cross-over situations, which in turn significantly reduces false positives.

When considering the degree of blur, in-focus objects are easily identified. However, when foreground objects become blurred, it makes the judgement of whether the objects are blur artefacts or genuine objects difficult, particularly with low intensity images. The definition of subspaces in this study takes this uncertainty into account. A low intensity pixel is given a small 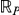 subspace, whereas a high intensity pixel is given a large 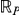 subspace.

When comparing the CICs and CBCs results using either control OSC data or pseudo-data from test cases 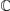 and 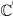, the differences between these two measurements are minor. Therefore, we consider CICs and CBCs to be equally robust. The calculation of CBCs is less computationally intensive and therefore recommended.

An innovative co-localisation measurement method is established in this study and a large quantity of cross-over incidences can now be identified and excluded from co-localisation quantification. Admittedly it will remain difficult to achieve a perfect co-localisation measurement due the complex nature of such images where noises, artefacts and biologically significant random partial overlap exist. Therefore for future works, the following three areas are worth addressing:

- Improvements and Automation: After Otsu's thresholding, the co-localisation mask *ASCI* can be manipulated using advance morphological operations, to i) move isolated small regions, ii) link broken lines, and iii) fill regions between the boundaries of myelin sheaths. Additionally as four parameters need to be set using the proposed method, the sensitivity of the choices of these parameters to noise, spherical aberration and axial under-sampling need to be studied, and an automated/guided parameter setting method would be beneficial to the reduction in operator subjectivities.
- Other Types of Samples: Though the results from both pseudo data and OSC data proves the robustness of our proposed method, it would certainly be beneficial to perform such an evaluation with data from not only the OSC but also other types of biological samples such as innervated peripheral tissue or vascular tumour tissue.
- Beyond the Z-Stack: In the context of the OSC data, the majority of foreground objects are axons and myelin sheaths, which form long and slender shapes in the xy-plane. When observing the orthogonal view, images predominantly represent transverse sections of neuronal structures. In this study we utilised neighbouring information from the z-stack direction, however refinement of this quantification method could also take into consideration additional biological information and image data characteristics presented in xy-planes.

For the sampled OSC data used in this study, a 40× oil immersion objective was used which gives a resolution of 0.25 µm/pixel. Not all CNS axons are myelinated, indeed only large diameter axons benefit from myelination in terms of improved conductance. As the diameter of unmyelinated axons in CNS tissue are in the range of between 0.08 and 0.4 µm [29], the majority of remyelinated axons would not be under-sampled based on a 2 pixel (0.5 µm) limit. However, it is important to note that for other types of biological studies, under-sampling could occur and thus appropriate imaging procedures would need to be selected to avoid under-sampling. However, our method of co-localisation may in fact be suitable for analysis of under-sampled images, though a comprehensive future study is needed to determine the influence of such sampling error on our method and other co-localisation measurements.

As far as the authors are aware of, almost all image based co-localisation measurement methods in the literature would be affected by changes in both labelling and imaging protocols. This represents one of the critical challenges in the community. It is important to follow strict quality control protocols throughout the sampling, labelling and imaging procedures to produce as little variation between experiments as possible. The proposed method in this study can be fine tuned with 4 input parameters to minimize such variation (see Parameter Selections from the Information S2 section). To get consistent results, it is advised that an experienced scientist should define these parameters for each batch of samples.

Although this method was designed for the purpose of measuring co-localisation of filamentous cellular processes of CNS tissue, there is also potential to utilise this method for other biological tissues and cells. The ability to recognise similarities of biological entitles in the z-direction can assist in identifying interaction between cell types in other physiological and pathological tissues such as tumour tissue. It can also be taken into account when measuring interaction between intracellular proteins and structures in high magnification microscopy. Therefore this relatively straightforward co-localisation measurement could be useful and impact on other areas of biological research that analyse confocal z axis imagery.

## Supporting Information

Information S1 **The Calculation of CICs and CBCs.**
(DOC)

Information S2 **Parameter Selections.**
(DOC)

## References

[1] Botti M, Gazza F, Ragionieri L, Acone F, Bo Minelli L (2005). Double Labelling Immunohistochemistry on the Paravertebral Ganglion Related to the Smooth Vasal Musculature of the Swine Cremaster Muscle.. Anatomia, Histologia, Embryologia.

[2] Riner K, Liesegang A, Boos A (2005). Vitamin D3 Receptor Immunohistochemistry in Sheep and Goat Intestine.. Anatomia, Histologia, Embryologia.

[3] Rodgers JL, Nicewander WA (1988). Thirteen Ways to Look at the Correlation Coefficient.. The American Statistician.

[4] Bolte S, CordeliÈRes FP (2006). A guided tour into subcellular colocalization analysis in light microscopy.. Journal of Microscopy.

[5] Manders EMM, Verbeek FJ, Aten JA (1993). Measurement of co-localization of objects in dual color confocal images.. Journal of Microscopy.

[6] Villalta JI, Galli S, Iacaruso MF, Antico Arciuch VG, Poderoso JJ (2011). New Algorithm to Determine True Colocalization in Combination with Image Restoration and Time-Lapse Confocal Microscopy to Map Kinases in Mitochondria.. PLoS ONE.

[7] Landmann L, Marbet P (2004). Colocalization analysis yields superior results after image restoration.. Microscopy Research and Technique.

[8] Wiseman PW, Squier JA, Ellisman MH, Wilson KR (2000). Two-photon image correlation spectroscopy and image cross-correlation spectroscopy.. Journal of Microscopy.

[9] Comeau JWD, Kolin DL, Wiseman PW (2008). Accurate measurements of protein interactions in cells via improved spatial image cross-correlation spectroscopy.. Molecular BioSystems.

[10] Wu Y, Eghbali M, Ou J, Lu R, Toro L (2010). Quantitative Determination of Spatial Protein-Protein Correlations in Fluorescence Confocal Microscopy.. Biophysical Journal.

[11] Lachmanovich E, Shvartsman DE, Malka Y, Botvin C, Henis YI (2003). Co-localization analysis of complex formation among membrane proteins by computerized fluorescence microscopy: application to immunofluorescence co-patching studies.. Journal of Microscopy.

[12] Costes SV, Daelemans D, Cho EH, Dobbin Z, Pavlakis G (2004). Automatic and Quantitative Measurement of Protein-Protein Colocalization in Live Cells.. Biophysical Journal.

[13] Li Q, Lau A, Morris TJ, Guo L, Fordyce CB (2004). A Syntaxin 1, Gαo, and N-Type Calcium Channel Complex at a Presynaptic Nerve Terminal: Analysis by Quantitative Immunocolocalization.. The Journal of Neuroscience.

[14] Landmann L (2002). Deconvolution improves colocalization analysis of multiple fluorochromes in 3D confocal data sets more than filtering techniques.. Journal of Microscopy.

[15] Fletcher PA, Scriven DRL, Schulson MN, Moore EDW (2010). Multi-Image Colocalization and Its Statistical Significance.. Biophysical Journal.

[16] Norton WT, Poduslo SE (1973). Myelination in rat brain: method of myelin isolation.. Journal of Neurochemistry.

[17] Ahrens J, Dieter U (1974). Computer methods for sampling from gamma, beta, poisson and bionomial distributions.. Computing.

[18] Zhou W, Zhang D (1999). Progressive switching median filter for the removal of impulse noise from highly corrupted images.. Circuits and Systems II: Analog and Digital Signal Processing, IEEE Transactions on.

[19] Lian J, Garner G, Muessig D, Lang V (2010). A simple method to quantify the morphological similarity between signals.. Signal Processing.

[20] Otsu N (1979). A Threshold Selection Method from Gray-Level Histograms.. Systems, Man and Cybernetics, IEEE Transactions on.

[21] Zhang H, Jarjour AA, Boyd A, Williams A (2011). Central nervous system remyelination in culture – A tool for multiple sclerosis research.. Experimental Neurology In Press, Corrected Proof.

[22] Stoppini L, Buchs PA, Muller D (1991). A simple method for organotypic cultures of nervous tissue.. Journal of Neuroscience Methods.

[23] Gonzalez RC, Woods RE (2002). Digital Image Processing (2nd Edition).

[24] Goh K-I, Cusick ME, Valle D, Childs B, Vidal M (2007). The human disease network.. Proceedings of the National Academy of Sciences.

[25] Steensel Bv, Binnendijk EPv, Hornsby CD, Voort HTvd, Krozowski ZS (1996). Partial colocalization of glucocorticoid and mineralocorticoid receptors in discrete compartments in nuclei of rat hippocampus neurons.. Journal of Cell Science.

[26] Manders EM, Stap J, Brakenhoff GJ, van Driel R, Aten JA (1992). Dynamics of three-dimensional replication patterns during the S-phase, analysed by double labelling of DNA and confocal microscopy.. Journal of Cell Science.

[27] Forutanpour B, WIPO (2010). Response to detection of Blur in an image.. H04N 5/232 (2006.01) ed.

[28] Tong H, Mingjing L, Hongjiang Z, Changshui Z

[29] Debanne D, Campanac E, Bialowas A, Carlier E, Alcaraz G (2011). Axon Physiology.. Physiological Reviews.

