# Supplementary material for: A Robust Co-Localisation Measurement Utilising Z-Stack Image Intensity Similarities for Biological Studies
Source: PLoS One. 2012 Feb 17;7(2):e30632. doi: 10.1371/journal.pone.0030632 (PMC3281864; doi:10.1371/journal.pone.0030632)
Supplement: Information S1 — The Calculation of CICs and CBCs. (DOC) [file pone.0030632.s001.doc]

Supporting Information S1: The Calculation of CICs and CBCs

This section describes the how to calculate the proposed Co-localisation Intensity Coefficients (CICs) and Co-localisation Binary Coefficients (CBCs), as well as explanations detailing why this method is used.

To differentiate co-localisation cases (e.g. Figure 1A) from exclusion cases (e.g. Figure 1B), we adopted the following method [1] which makes use of data generated from the same pixel across focal planes in the z-stack.

Given a set of *n* neighbouring focal planes *P*=[*P_1_*,*P_2_*,…,*P_n_*] which are captured as digital images, observation suggests that a non-background pixel with the coordinates of (*x*,*y*) in a random focal plane *P_k_* from either *C_α_* or *C_β_*, when the microscope focal lens is fine tuned, the appearance of this pixel at (*x*,*y*) changes from blurred to focused (sharp) and then to blurred again along the z-stack. According to the point spread function (PSF) [2], the light emission for a sharp object would in fact spread extending to infinity in the z-axis direction. However in digitally captured images, this light spread (blur) is only visible/detectable in a short distance range when the movements of the lens are close to the height Ψ of the foreground 3D object. The blur artefacts are in fact the changes of image intensity *I* at location (*x*,*y*) across all in range focal planes, where *I*=[*I_1_*,*I_2_*,…,*I_k_*_-2_,*I_k_*_-1_,*I_k_*,*I_k_*_+1_,*I_k_*_+2_,…,*I_m_*] and *m* is number of focal planes captured within the range of Ψ. When the value of *m* is small, e.g. less than or equal to 5 focal planes, interpolation can be used to generate more values for vector *I*. Padding is used at the top and bottom of the z-stack, e.g. when dealing with the first focal plane *P_1_*, we are considering the neighbouring pixel intensities [*I_1_*,*I_1_*,*I_1_*,*I_2_*,*I_3_*] which simply replicated the value of *I_1_* twice.

Initially, three subspaces are defined in the image intensity domain. Given the image intensity space of within the range of [0,255] or [0,1] after normalisation, is subdivided into three subspaces namely the positive subspace , zero subspace and negative subspace , where . These three subspaces are subsequently used as guidelines to define if a random pixel at location (*x*,*y*) across *C_α_* or *C_β_* colour channels are similar. Therefore, the degree of pixel similarity is dependent on the definition of subpaces , and .

In our experiments, the three subspaces , and are defined to be linearly related to the value of image intensity. To use a vector of image intensities at location (*x*,*y*) across *m* focal planes in colour channels *C_α_*,

$I^{\alpha}=\left[I_{1}^{\alpha},I_{2}^{\alpha},\ldots,I_{q}^{\alpha}\right]$ (1)

where *q*≥*m*. If *q*>*m*, *I^α^* is in fact the interpolated image intensity data. Four boundary lines *U_d_*, *L_d_*, *U_p_* and *L_p_* are defined as:

(2)

(3)

(4)

(5)

where *ε*_1_, *ε*_2_$\in$[0,1]. These four boundary lines are then used to partition the image intensity space of into the three image intensity subspaces , and . Given a random image intensity value $\hat{I}$, , and are defined as

(6)

(7)

(8)

A number of examples of the partition of three intensity subspace are shown in the Results and Evaluation Section (Figure 9E-F, 9B-I & 10). The region between *L_p_* and *U_p_* is the positive subspace , the region between *L_d_* and *L_p_* as well as *U_p_* and *U_d_* is defined as the zero subspace , and the negative subspace is the region smaller than *L_d_* as well as the region bigger than *U_d_*.

The next step is signal trichotomisation. The image intensity is defined in the space of with the value of [0,255] or [0,1] after normalisation. is then subdivided into three subspaces namely positive subspace , zero subspace and negative subspace , where . The trichotomisation is defined as

(9)

If two colour channels, *C_α_* and *C_β_*, are considered, given the trichotomisation of *t_α_*(*x*,*y*,*k*) and *t_β_*(*x*,*y*,*k*), their signed product is defined as

(10)

where is the signed product operator.

The signed correlation of the two image intensity signal is given by

$T_{\alpha,k}\left(x,y\right)\circ T_{\beta,k}\left(x,y\right)=\sum_{k=1}^{m} t_{\alpha}\left(x,y,k\right)\otimes t_{\beta}\left(x,y,k\right)$ (11)

where is the signed correlation.

The signal similarity measurement *ASCI* between colour channel *C_α_* and *C_β_* is defined as

(12)

*ASCI_α,β,k_*(*x*,*y*) describes the degree of similarity between two image intensity vectors at pixel location (*x*,*y*) across *C_α_* or *C_β_* colour channels. The value of *ASCI_α,β,k_*(*x*,*y*) is in the range of [-1,1]. The general rule is that when values of the two intensity vectors are morphologically very similar, the value of *ASCI_α,β,k_*(*x*,*y*) approaches 1. The value of *ASCI_α,β,k_*(*x*,*y*) is lower and approaches -1 when the values of the two intensity vectors are morphologically different.

Two foreground pixels at a same location (*x_l_*,*y_l_*) and a same focal plane *P_k_* from two colour channels (*C_α_*,*C_β_*) are considered to be co-localised if

$ASCI_{\alpha,\beta,k}\left(x_{l},y_{l}\right)>\rho$ (13)

where *l*=1,2,…,*L*, and *L* is the number of co-localised pixels from focal plane *P_k_*. As the value of *ASCI_α,β,k_*(*x*,*y*) is in the range of [-1,1], the threshold value of *ρ* is set to be 0. Therefore, a positive value for *ASCI_α,β,k_*(*x*,*y*) indicates the potential existence of co-localisation whereas a negative or zero value of *ASCI_α,β,k_*(*x*,*y*) suggests exclusion. It is noted that among all the *L* co-localised pixels, a subset in *L* represents background pixels. Though these background pixels are co-localised, they are to be removed in the next step.

As described in the Methodology section, all background pixels from focal plane *P_k_* for both colour channel *C_α_* and *C_β_* are then recognised and removed using Otsu’s global thresholding method [3]. For each colour channel, a single threshold value is generated which uses all image pixel values across the whole stack of focal planes to define such a threshold value.

After Otsu’s thresholding, a total of *J* foreground pixels remain for focal plane *P_k_*, and $\hat{L}$ co-localised pixels left, whereas $\hat{L}\in L$. All background pixels which were considered co-localised are removed.

Similar to the definition of Co-localisation Coefficients [4], the proposed Co-localisation Intensity Coefficient (CIC) for a given focal plane *P_k_* is defined as

(14)

References

1. Lian J, Garner G, Muessig D, Lang V (2010) A simple method to quantify the morphological similarity between signals. Signal Processing 90: 684.
2. Kimura S, Munakata C (1989) Calculation of three-dimensional optical transfer function for a confocal scanning fluorescent microscope. J Opt Soc Am A 6: 1015.
3. Otsu N (1979) A Threshold Selection Method from Gray-Level Histograms. Systems, Man and Cybernetics, IEEE Transactions on 9: 62.
4. Manders EMM, Verbeek FJ, Aten JA (1993) Measurement of co-localization of objects in dual color confocal images. Journal of Microscopy 169: 375-382.
