# Supplementary material for: A Robust Co-Localisation Measurement Utilising Z-Stack Image Intensity Similarities for Biological Studies
Source: PLoS One. 2012 Feb 17;7(2):e30632. doi: 10.1371/journal.pone.0030632 (PMC3281864; doi:10.1371/journal.pone.0030632)
Supplement: Information S2 — Parameter Selections. (DOC) [file pone.0030632.s002.doc]

Supporting Information S2: Parameter Selections

This proposed method is simple in principle and easy to use and understand. Four parameters can be used to fine tune the results, which are *ε*_1_, *ε*_2_, *ρ* and *m*, In our experiments with the remyelination data, pseudo test dataset and , the following parameter settings were used unless specified otherwise: *m*=9, *ε*_1_=0.5, *ε*_2_=0.5 and *ρ*=0.

Parameter *ε*_1_ and *ε*_2_ are used to define the four boundary lines *U_d_*, *L_d_*, *U_p_* and *L_p_* which are subsequently used for partition of image intensity space into , and . *ε*_1_ and *ε*_2_ can choose any values in the range of [0,1]. As a rule of thumb, for images with considerable noise, larger values (>0.5) of *ε*_1_ and *ε*_2_ should be chosen. With larger *ε*_1_ and *ε*_2_ values, the range of the positive and zero intensity space and are enlarged, so that potential co-localised pixels are not missed. The amount of false co-localised pixels introduced is trivial as it is unlikely for pixels at location (*x*,*y*) to be noisy across a continuous number of focal planes. For images with less noise, our experiments suggest the value of *ε*_1_=0.5 and *ε*_2_=0.5 would be appropriate.

Parameter *ρ* is used to define if the intensity similarity measurement *ASCI_α,β,k_*(*x*,*y*) is satisfied at location (*x*,*y*) on focal plane *k* across the two colour channel *C_α_* and *C_β_*. By assuming that *ASCI_α,β,k_*(*x*,*y*) is linearly distributed within the scope of [-1,1], we discard all the values for *ρ*≤0 as not co-localised, and only consider *ρ*>0 cases to be potentially co-localised.

Parameter *m* is the count for the number of focal planes which determines how many neighbouring planes should be considered when calculating the intensity similarity measurement *ASCI_α,β,k_*(*x*,*y*). The value for *m* should be set to be as close as possible to the number of focal planes that the average height of a foreground object would cover. This can be easily achieved by viewing through the stack of data and counting the number of focal planes (depending on the intervals among focal planes) from the appearance to the disappearance of a foreground object. A smaller *m* value would typically increase the false positives because exclusion regions incorrectly recognised as co-localisation as shown in case 3 and 4 in Figure S1. Whereas a larger *m* value would increase the false negatives (co-localised regions incorrectly regarded as exclusion) as shown in case 1 and 2 in Figure S1.


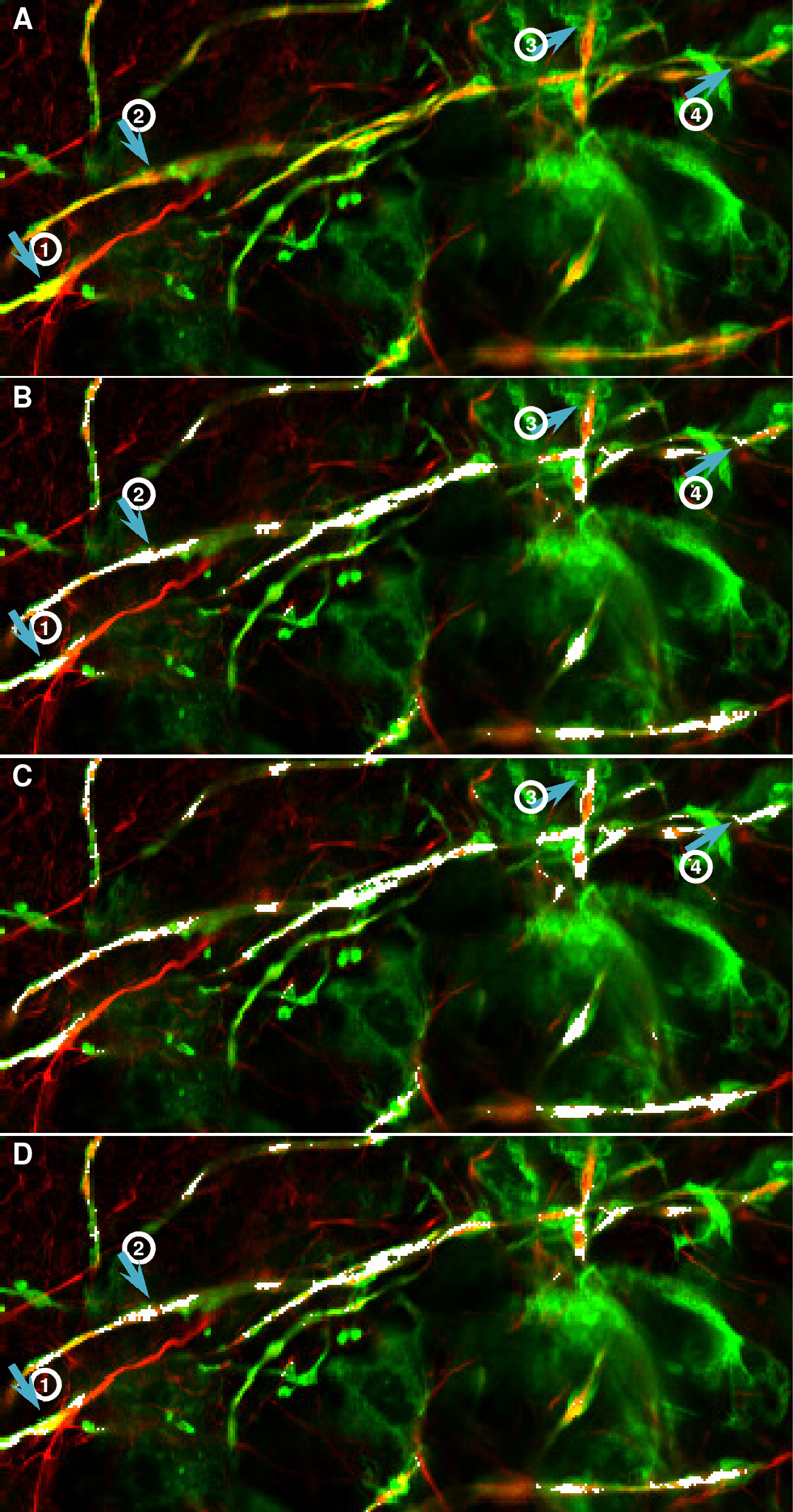


Figure Legends

Figure S1. Illustration of the effect of increased false positives and false negatives using incorrectly selected *m* value using murine organotypic brain slice cultures.

Figure S1 detailed legend: (A) An example of a focal plane from murine OSC at 40X magnification, (B) the calculation of co-localisation using *m*=9 (the correct choice), (C) the calculation of co-localisation using a small *m* value, *m*=3, which increased the false positives (indicated with arrow 3 and 4), (D) the calculation of co-localisation using a large *m* value, *m*=17, which increased the false negatives (indicated with arrow 1 and 2), *In figure B-D, co-localised regions are marked in white.
